# Effectiveness of the maternal and child health handbook for improving continuum of care and other maternal and child health indicators: A cluster randomized controlled trial in Angola

**DOI:** 10.7189/jogh.13.04022

**Published:** 2023-02-03

**Authors:** Olukunmi Omobolanle Balogun, Ai Aoki, Caroline Kaori Tomo, Keiji Mochida, Sachi Fukushima, Masashi Mikami, Toru Sadamori, Michiru Kuramata, Helga Reis Freitas, Pedro Sapalalo, Lino Tchicondingosse, Rintaro Mori, Hirotsugu Aiga, Ketha Rubuz Francisco, Kenji Takehara

**Affiliations:** 1 Department of Health Policy, National Center for Child Health and Development, Tokyo, Japan; 2 TA Networking Corp., Tokyo, Japan; 3 Department of Global Health, Graduate School of Health Sciences, University of the Ryukyus, Nakagami, Okinawa, Japan; 4 Department of Data Science, Biostatistics Unit, Clinical Research Center, National Center for Child Health and Development, Tokyo, Japan; 5 Samauma Consulting LLC, Tokyo, Japan; 6 Department of Primary Healthcare, National Directorate of Public Health, Ministry of Health, Luanda, Angola; 7 Domus Custodius (SU) Lda. Tchikos Agency, Luanda, Angola; 8 Graduate School of Medicine, Kyoto University, Kyoto, Japan; 9 School of Tropical Medicine and Global Health, Nagasaki University, Nagasaki, Japan; 10 Department of Human Development, Japan International Cooperation Agency, Tokyo, Japan

## Abstract

**Background:**

The maternal and child health (MCH) handbook is promoted as a tool for strengthening continuum of care. We assessed the effect of a MCH handbook intervention package on continuum of maternal and child health care and health outcomes for mother and child.

**Methods:**

We conducted an open-label, parallel two-arm cluster randomized controlled trial in Angola. We randomly assigned municipalities in Benguela province through block randomization to a group using a package of enhanced maternity care service (which included the MCH handbook distribution and its supplementary intervention) and another using usual care (two stand-alone home-based records). We included women who were pregnant at the beginning of the trial period and attended a public health care facility for maternity care services. Neither health care providers, study participants nor data assessors were masked, but the statistician was. The primary outcome was a measure of service utilization assessed via achievement of maternal behavior-based continuum of care at three months postpartum. We conducted an intention-to-treat analysis in women with available data.

**Results:**

We randomized 10 municipalities to either the intervention (five clusters) or control (five clusters) group. Of the 11 530 women approached between June 8, 2019, and September 30, 2020, 11 006 were recruited and 9039 included in the final analysis (82%; 3774 in the intervention group and 5265 in the control group). The odds for achievement of maternal behavior-based continuum of care in the intervention group was not significantly different from that in the control group (adjusted odds ratio (aOR) = 1.18, 95% confidence interval (CI) = 0.46-2.93) at three months postpartum. However, the odds of initiating antenatal care clinic use were significantly higher in the intervention group (odds ratio (OR) = 5.16, 95% CI = 2.50-10.67). No harms associated with the intervention were reported.

**Conclusions:**

Distribution of the MCH handbook and its supplementary interventions promoted initiation of antenatal care service use, but did not increase service utilization sufficiently enough for attainment of study defined maternal behavior-based continuum of care.

**Registration:**

ISRCTN20510127.

The maternal and child (MCH) handbook is an integrated home-based record and is part of a scheme designed to record in a single document, all the information regarding health services provided to a mother, and the health conditions of her and her child, in a single document [1,2]. The MCH handbook covers all the stages of maternal, newborn, and child health (MNCH) from antenatal care to delivery, postnatal care, child vaccinations and growth monitoring, and provides health information to parents [1]. Alongside other uses, the MCH handbook has the potential to reduce the need for multiple health records [3] and provide support for improvements in continuum of care (CoC) [4,5]. Consequently, the MCH handbook has been attracting more attention from health ministries and professional organizations as an effective tool for protecting the health of mothers and children, and promoting a life course approach to health care [2].

Although the World Health Organization (WHO) recommends the use of home-based records as complementary to facility-based records, the superiority of the MCH handbook over other home-based record formats remains unclear [6,7]. Furthermore, there are few high-quality studies on MCH handbook use [7], especially from sub-Saharan African countries that potentially may benefit more from its use as a primary record keeping and instructional tool maintained at home than other countries [6]. To address some of the research gaps highlighted by WHO guideline for home-based records [6,7], we conducted a cluster randomized controlled trial comparing the use of the integrated MCH handbook with the use of two stand-alone home-based records in Angola.

The Republic of Angola is a lower middle-income level country located along the west coast of Southern Africa [8]. Despite substantial economic and political progress made in recent years, health sector development varied within the country and health indicators are yet to reach adequate standards. The 2015-2016 Demographic and Health Survey data from Angola show that 239 maternal deaths occurred per 100 000 live births; while neonatal and infant mortality rates were 24 and 44 deaths per 1000 live births respectively [9]. One of the factors contributing to the poor maternal and child health indicators is the weakness of health management systems [10]. Additionally, a large information gap on health indicators makes it difficult to inform the decision-making process in the country [11].

The Government of Angola’s National Plan for Health Development 2012-2025 prioritizes pregnant women, infants, and young children as target populations; and the government, with support from the United Nations (UN), Japan International Cooperation Agency (JICA), and other organizations, is working to strengthen the health information system in the collection, processing, and analysis of data [10]. Thus, the Angolan Ministry of Health, in partnership with JICA, developed and implemented the MCH handbook program to improve record keeping and promote maternal and child health care service utilization. We aimed to evaluate the impact of an intervention package involving the use of the MCH handbook and its supplementary interventions, compared with traditional use of two stand-alone home-based records on MNCH service utilization from pregnancy through the postnatal and early childhood period among women in Angola.

## METHODS

### Study design and location

This study was an open-label, parallel two-arm cluster randomized controlled trial done within a pregnancy cohort in which the use of the MCH handbook was compared with the use of two stand-alone home-based records during maternity and childcare service use in Angola. We purposively selected the Benguela province, as its health indicators are close to the national average, thus representing an ideal setting to show intervention impact on MCH outcomes and demonstrate the feasibility for a nationwide scale-up of the MCH handbook in Angola. We favored the cluster randomization design due to the nature of the intervention and because it minimized logistical and scientific difficulties associated with individual randomization in large-scale longitudinal studies. The central study center in Angola was based at the Benguela office of Domus Custodius Holding TCHIKOS Consulting Agency, and the overall study coordinating center was at the National Center for Child Health and Development, Tokyo Japan.

### Participants

All public health care facilities providing MNCH services in Benguela province were eligible. Our pregnancy cohort included women who were pregnant at the beginning of the study period and who sought MNCH services in participating health care facilities. Women were eligible for inclusion if they had their last menstrual period between March 1 to April 30, 2019, or probable delivery from December 1, 2019, to January 31, 2020, providing they were also not participating in another study, not planning to move out of the study area during the study period, and that they gave written informed consent. We recruited participants across the MNCH service spectrum during their first health care facility visit during the index pregnancy for any of antenatal care, delivery, postnatal care, or, child vaccination services over the study period.

### Randomization and masking

This was a two-arm study comprising an intervention group and a control group; while a cluster was defined as one municipality. We allocated the municipalities to either intervention or control groups using block randomization (shown in Figure S1 in the **Online Supplementary Document**) conducted by the study statistician, as previously described [12]. The statistician was unable to predict assignment to either study group. All health care facilities located in the intervention group were service delivery points for the intervention package, while facilities located in the control group delivered the usual care. We assigned women to either study group depending on the location of the health care facility where they attended their first MNCH service. Masking was not possible due to the nature of the intervention; thus, neither participants, health care providers, data collectors at each facility, nor the rest of the study team were masked. Only the data analyst was masked to cluster assignment. Data masking was achieved by replacing municipality names with numbers and study groups with letters.

### Intervention

We based our study rationale on research gaps highlighted in the 2018 WHO recommendations on home-based records for maternal, newborn, and child health [6,7]. The guidelines stressed the lack of knowledge on the usefulness and appropriateness of the integrated health handbooks and called for research on whether the MCH handbooks are superior to stand-alone home-based records, and if so, under what conditions [6]. Subsequently, we administered our intervention package after the MCH handbook was designed through the MCH handbook committee comprising members from the Ministry of Health in Angola, the WHO, UNICEF, JICA, and local bodies (including the Angolan Pediatric Society). The MCH handbook development process in Angola has been previously reported [12,13]. The study design identified in the formative phase involved a community-wide distribution of the MCH handbook with its supplementary interventions.

Study interventions leveraged existing health care infrastructure to facilitate intervention delivery, which was divided into three parts: 1) distribution of MCH handbook; 2) health care provider training on MCH handbook operation, and 3) community sensitization and mobilization among pregnant women on the use of the MCH handbook. We provided the MCH handbook to women during their first contact with health care facilities to receive MNCH services for the index pregnancy, with explanations on when and how to use the MCH handbook and on data recording. Women also received health education based on the contents of the MCH handbook during routine prenatal classes. Women in the control group continued to benefit from the traditional use of two stand-alone home-based records – the prenatal card and child health card. In both study groups, we asked women to bring their MCH handbook or prenatal card and child health card to every occasion of MNCH service consultation. We trained health care providers on MCH handbook operation prior to distribution of the MCH handbook to pregnant women/mothers. Initially, we provided a five-day training of trainers for selected health care professionals from participating facilities in the intervention group. Subsequently, trainers from each health care facility provided on-the-job training to health care providers at their respective facilities. The training focused on fundamentals of MNCH services, how to use and record information in the MCH handbook during regular consultations, how to explain the use of the MCH handbook to pregnant women/mothers, and how to interpret data entries from the MCH handbook. To enhance eligibility assessment, health care providers in both study groups received training on interviewing techniques for determining the date of the last menstrual period and on methods for calculating probable delivery date. We provided in-facility refresher training of trainers if required following periodic visits by the study team to intervention group facilities. Community sensitization and mobilization involved the provision of community events and mothers’ classes using the MCH handbook material as educational material.

### Procedures

Data collectors stationed at each health facility recruited the cohort participants, after which they collected data at each participant’s home or location of choice. The baseline survey included questions on socio-demographic factors, household characteristics, pregnancy history, maternal health behavior, and home-based record ownership and use. We suspended trial activities for four months due to COVID-19 lockdown restrictions in Angola, so we consequently extended the follow-up period from three months after delivery to six months postpartum and changed some outcome measures. We registered all changes in the trial registry prior to the end of data collection. We collected data regarding antenatal care attendance, maternal health behavior, pregnancy outcomes, infant feeding practices and infant and maternal health retrospectively from women during the follow-up survey. We double-entered the verbal responses obtained from mothers, with record entries from individual home-based records for antenatal care, delivery, and vaccination service use, when available. We collected the data digitally using interviewer-administered structured questionnaires delivered using the Open Data Kit (ODK) Collect application [14]. We matched the women’s responses from baseline interview to follow-up records using unique participant identification common to both data sets. Two researchers independently performed a case-by-case review of mismatched records using additional demographic variables.

### Outcomes

The primary outcome was a composite measure of maternal behavior-based CoC, including a minimum number of antenatal care visits, facility-based delivery, postnatal care for mother and newborn, and at least two child vaccinations clinic visits – at birth and at three months infant age. We followed- up the participants at 16 months after the first enrolment to enable outcome assessment for a continuum of maternal and child care past three months postpartum. Secondary outcomes were: a composite measure for achievement of service-based CoC at three months postpartum; frequency of MNCH service use; neonatal and infant mortality rates; infant health check-up; morbidity detection rate for mothers and infants; postpartum depression; maternal health behavior; infant feeding practices and child vaccination. Vaccinations received and date of vaccinations were obtained from home-based records used in the respective study groups. As per reporting guidance [15], we reported each component of the composite primary outcome as a secondary outcome. A detailed description of each outcome is provided in the study protocol [12].

### Statistical analyses

Applying a simulation-based power analysis using generalized linear mixed effect model, we estimated that a study with at least 10 000 participants would have 80% power to detect an estimated 10% difference in the primary outcome between groups with a two-sided α of 0.05 and an intra-cluster correlation coefficient of 0.01 (Table S1 in the **Online Supplementary Document**) [12]. We analyzed individual level data according to the study group to which women were originally allocated. We also planned per-protocol analysis [12] to examine intervention effects based on treatment received. However, due to very high agreement between the intention-to-treat analysis set and the per-protocol analysis set (presented in Table S2-3 in the **Online Supplementary Document**), we conducted only an intention-to-treat analysis.

We descriptively compared baseline characteristics between groups to check that randomization produced broadly similar groups. We conducted a single imputation by worst observation carried forward for missing values of binary outcome measures to maintain a conservative approach to the analysis. We conducted the primary and secondary outcome analyses with generalized linear mixed models assuming logit link and binomial distribution for binary outcomes, and cumulative logit link and multinomial distribution for multinomial and ordinal outcomes. The fixed effects were study group (intervention/control) and municipality location (urban/rural). We assumed clustering at the municipality level within the province in a fully nested framework and set municipalities to the random effect. We expressed effectiveness estimates as odds ratios (ORs) with 95% confidence intervals (CI) and reported using the Consolidated Standards of Reporting Trials (CONSORT) statement extended for cluster randomized controlled trials [16]. We conducted a subgroup analysis by facility location for the primary outcome and CoC achievement measures. For the statistical analyses, we used the SAS software version 9.4 (SAS Institute, Cary, NC, USA). We registered the trial with the ISRCTN Registry (ISRCTN20510127) where the full protocol is publicly available.

## RESULTS

In April 2019, we randomized 10 municipalities in Benguela province to either the intervention or control group. We required public health care facilities within each group to provide either standard MNCH services plus distribution of MCH handbook and its supplementary interventions (intervention group) or standard MNCH services with continued use of two stand-alone home-based records (control group). We started recruiting participants on June 8, 2019 and involved 190 public facilities (89 from intervention group and 101 from control group) in this study (Table S4 in the **Online Supplementary Document**). Details of facility types and number of participants recruited from each facility type are shown in Table S5 in the **Online Supplementary Document**. We included women who delivered between November 2019 and February 2020. We conducted follow-up surveys from August 3, 2020, to September 30, 2020, after COVID-19 restrictions in Angola were lifted.

**Figure 1:**
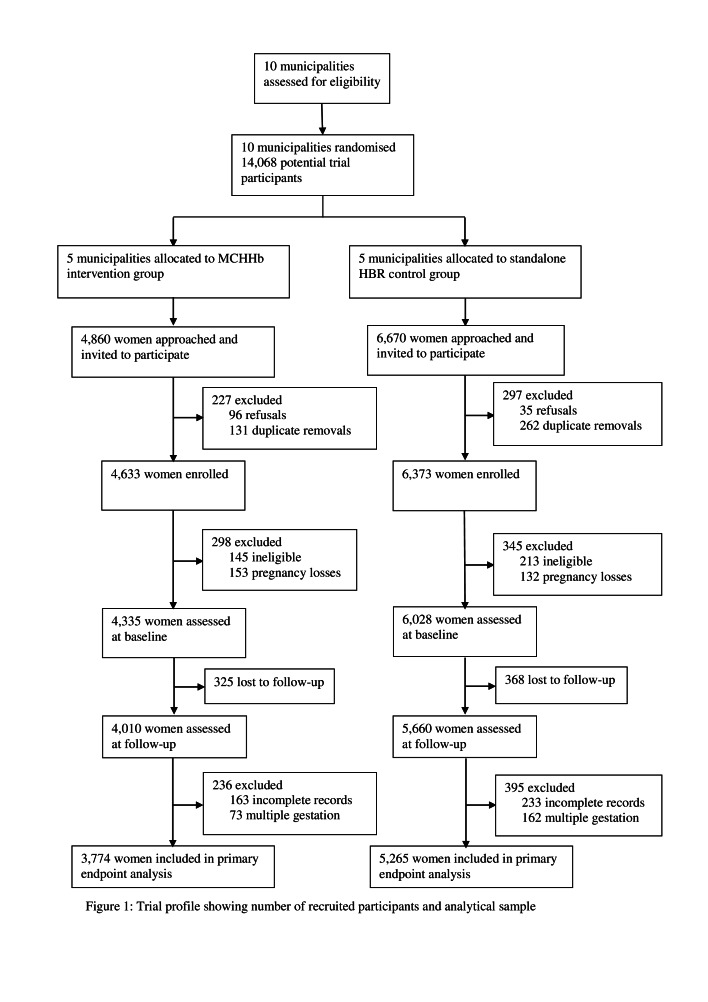
Trial profile showing number of recruited participants and analytical sample.

We approached a total of 11 530 women and invited them to participate in the study; 131 (1%) declined and 765 (7%) were approached multiple times, after which their duplicate records were removed, leaving 11 006 (95%) enrolled participants (4633 in the intervention group and 6373 in the control group) (**Figure 1**). We excluded 643 (6%) after the baseline interviews (358 with pregnancy outside eligibility period; 285 lost their pregnancies) and 631 (6%) after follow-up interviews (396 with no baseline data and 235 women with multiple births). At the end of the exit interviews, 693 (6%) were lost to follow-up and the final analysis included 9039 (82%; 3774 in the intervention group and 5265 in the control group) matched baseline and follow-up enrollee records (**Figure 1**). Maternal baseline characteristics were similar in both groups, but the post-hoc intra-cluster correlation coefficient was estimated to be about 0.04, larger than the initially assumed value of 0.01. Municipalities in the control group were more urban (**Table 1**) compared with those in the intervention group, so we adjusted for location in all subsequent analyses.

**Table 1:** Baseline characteristics of study participants

General characteristics	Intervention arm (n = 3774)	Control arm (n = 5265)	*P-*value
**Mean maternal age in years**	25 (14-45)	25 (14-45)	0.38
≤19	889 (24%)	1180 (22%)	0.86
20–24	1155 (31%)	1446 (27%)	
25–29	843 (22%)	1208 (23%)	
30–34	509 (13%)	769 (15%)	
≥35	316 (8%)	484 (9%)	
**Marital status**			
Married/cohabitation	2572 (68%)	3368 (64%)	0.31
Single	1107 (29%)	1729 (33%)	
Divorced or widowed	49 (1%)	40 (1%)	
**Education level**			
No formal education	775 (21%)	788 (15%)	0.88
Primary education	1441 (38%)	1702 (32%)	
Secondary education	1430 (38%)	2475 (47%)	
Higher education	62 (2%)	114 (2%)	
**Literacy**			
Literate	1805 (48%)	3158 (60%)	0.33
Illiterate	1915 (51%)	1928 (37%)	
**Living setting**			
Urban	1384 (37%)	3481 (66%)	<0.001
Rural	2390 (63%)	1784 (34%)	
**Employment**			
Housewife	1796 (48%)	2050 (39%)	0.74
Self-employed	1304 (35%)	1840 (35%)	
Public/private sector employee	192 (5%)	377 (7%)	
Student only	416 (11%)	795 (15%)	
**Household wealth index**			
Poorest	903 (24%)	852 (16%)	0.98
Poor	718 (19%)	636 (12%)	
Average	694 (18%)	930 (18%)	
Wealthy	560 (15%)	1054 (20%)	
Wealthiest	538 (14%)	1024 (19%)	
**Ethnicity**			
Umbundu	3319 (88%)	4442 (84%)	0.86
Other tribes	389 (10%)	667 (13%)	
Foreigners	4 (<1%)	8 (<1%)	
**Maternity and antenatal care**			
Parity			
*Primipara*	897 (24%)	1368 (26%)	0.29
*Multipara*	2832 (75%)	3766 (72%)	
Livebirths	3767 (99%)	5260 (99%)	0.39
Mean gestation age at booking, weeks	23 (4-43)	22 (4-43)	0.90
*≤20*	1442 (38%)	1976 (38%)	0.64
*21-30*	1717 (46%)	1977 (38%)	
*31-37*	458 (12%)	536 (10%)	
*≥38*	61 (2%)	159 (3%)	
No of ANC attended			
*At least one*	3681 (98%)	4655 (88%)	0.02
*<4*	1897 (50%)	1917 (36%)	0.37
*≥4*	1781 (47%)	2731 (52%)	
Type of service sought at recruitment			
*Antenatal care*	3200 (85%)	4039 (77%)	0.35
*Delivery*	6 (<1%)	70 (1%)	
*Postnatal care and family planning*	2 (<1%)	35 (1%)	
*Vaccination*	566 (15%)	1121 (21%)	
**HBR possession and use**			
Possession of prenatal card from previous pregnancy			
*Had prenatal card*	1082 (29%)	1459 (28%)	0.91
*No prenatal card*	1602 (42%)	2246 (43%)	
Possession of HBR for index pregnancy			
*Any HBR ownership*	3370 (89%)	4710 (89%)	0.96
*Ownership of MCH handbook*	3353 (89%)	1032 (20%)	0.01
*Prenatal card ownership*	293 (8%)	3412 (65%)	0.01
*Child health card ownership*	811 (21%)	4452 (85%)	0.01
*Owns both HBR*	833 (22%)	1013 (19%)	0.001
Carried HBR at consultations	2845 (75%)	4165 (79%)	0.63

ANC – antenatal care, HBR – home-based record, MCH – maternal and child health

**Figure 2:**
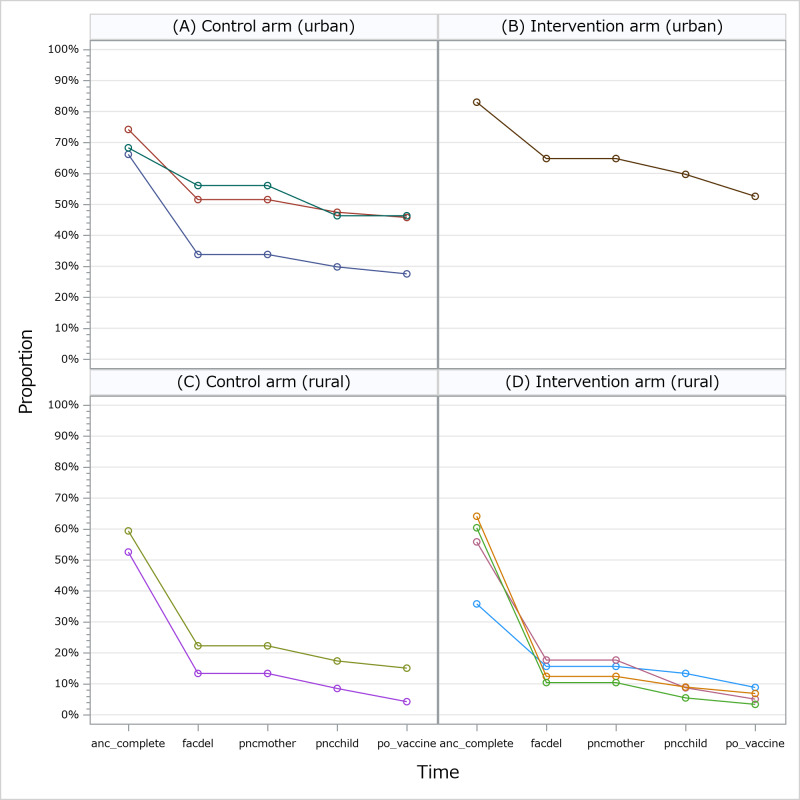
Changes in individual components of maternal behavior-based CoC in the intervention and control groups by municipality location (urban and rural). **Panel A**: proportion of women who achieved optimal MNCH service use in urban locations in control arm. **Panel B**: proportion of women who achieved optimal MNCH service use in urban locations in intervention arm. **Panel C**: proportion of women who achieved optimal MNCH service use in rural locations in control arm. **Panel D**: proportion of women who achieved optimal MNCH service use in rural locations in intervention arm. anc_complete – study defined optimal number of antenatal care visits, facdel  – delivery at health facility, pncmother – postnatal care for mother, pncchild – postnatal care for child, po_vaccine – study defined vaccination service use achievement.

At three months postpartum, the odds for achievement of maternal behavior-based CoC in the intervention group were not significantly different from those of the control group (1132 (30%) of 3774 women in the intervention group vs 2145 (41%) of 5265 in the control group; OR = 1.18; 95% CI = 0.48-2.93 (**Table 2**)). The proportion of women who attained the minimum required number of antenatal care service use was similar across the study groups. Facility-based delivery and the use of postnatal care services were comparable between both study groups, while women in the intervention group were less likely to have attended infant postnatal care or achieve at least two vaccination clinic visits as at three-month infant age. Changes in individual components of the primary outcome by area are presented in **Figure 2**. Unlike in the urban areas, there was a steep decrease in the number of facility-based deliveries in rural areas even among women who attained the required minimum antenatal care service use. We observed a similar trend in both intervention and control groups, with a slightly steeper drop in the intervention group.

**Table 2:** Effect of the intervention on continuum of care up to three months postpartum

	Intervention arm (n = 3774)	Control arm (n = 5265)	OR (95% CI)
**Primary outcome**			
Maternal behavior-based CoC	1132 (30%)	2145 (41%)	1.18 (0.48-2.93)
**Secondary outcomes**			
Minimum expected number of ANC	2540 (67%)	3628 (69%)	1.27 (0.65-2.49)
Facility based delivery	1945 (52%)	3380 (64%)	1.06 (0.58-1.93)
Postnatal care for mother	2045 (54%)	3537 (67%)	1.00 (0.52-1.94)
Postnatal care for baby	2446 (65%)	4149 (79%)	0.85 (0.37-1.95)
Two vaccination clinic visits at three months	1913 (51%)	3782 (72%)	0.69 (0.28-1.68)
**Service-based CoC**	990 (26%)	1998 (38%)	1.06 (0.32-3.53)
Seven vaccinations received at three months	1415 (37%)	3294 (63%)	0.67 (0.16-2.71)

CoC – continuum of care, ANC – antenatal care, OR – odds ratio, CI – confidence interval

Compared to maternal behavior-based CoC, a smaller proportion of women achieved service-based CoC in both groups (26% vs 30% in the intervention group and 38% vs 41% in the control group) and the probability of having received all seven childhood vaccinations was lower among women in the intervention group compared to those in the control group (**Table 2**). Results of subgroup analyses for CoC achievement by facility location showed no significant difference between the groups (presented in Table S6 in the **Online Supplementary Document**).

Findings on the secondary outcomes reflected a similar trend, except for antenatal care coverage of at least one visit. The odds of attending at least one antenatal care clinic was significantly higher in the intervention group compared to the control group (OR = 5.16, 95% CI = 2.50-10.67) (**Table 3**). The MCH handbook intervention package showed no significant differences between groups on other secondary outcomes. In the intervention group, a lesser proportion of women reported alcohol use during pregnancy, while a greater proportion of women reported attending HIV counselling and testing for PMTCT (**Table 3**), however the differences were not statistically significant. Regarding other secondary outcomes, no statistically significant differences were observed in the proportion of women who reported neonatal or infant deaths, infant health check-ups, maternal morbidities and pregnancy complications, infant feeding practices, and complete vaccination at six months infant age. No harms associated with the intervention were reported.

**Table 3:** Effect of the intervention on continuum of care up to six months postpartum

Secondary outcomes	Intervention arm (n = 3774)	Control arm (n = 5265)	OR (95% CI)
**Neonatal mortality**	105 (3%)	89 (2%)	1.38 (0.66,2.89)
**Frequency of ANC service use**			
At least one	3681 (98%)	4655 (88%)	5.16 (2.50-10.67)
≥4	1781 (47%)	2731 (52%)	0 · 94 (0.50-1.77)
≥8	78 (2%)	203 (4%)	0 · 47 (0.07-3.09)
**Infant health check**	2297 (61%)	4094 (78%)	0 · 79 (0.29-2.18)
**Maternal morbidities and pregnancy complications detected**			
Stillbirth	7 (<1%)	5 (<1%)	1.77 (0.18-17.43)
HIV	306 (8%)	550 (10%)	0.49 (0.17-1.39)
Anaemia	302 (8%)	318 (6%)	1.32 (0.43-4.04)
**Infant morbidity**	1212 (32%)	1844 (35%)	0.78 (0.45-1.35)
**Infant mortality**	204 (5%)	191 (4%)	1.17 (0.55-2.49)
**Maternal health behavior**			
Current alcohol use	157 (4%)	349 (7%)	0.76 (0.29-1.96)
Current tobacco use	56 (1%)	78 (1%)	1.19 (0.26-5.51)
PMTCT	2128 (56%)	2588 (49%)	1.43 (0.62-3.34)
Family planning use	337 (9%)	866 (16%)	0.64 (0.17-2.41)
Correct knowledge alcohol use	3145 (83%)	4675 (89%)	0.70 (0.16-3.20)
Correct knowledge tobacco use	3108 (82%)	4678 (89%)	0.70 (0.15-3.27)
**Malaria prevention**			
IPTp	1125 (30%)	1672 (32%)	1.26 (0.80-1.98)
**Maternal depression**	1234 (33%)	1523 (29%)	0.68 (0.23-2.05)
**Infant feeding practices**			
Early initiation of breastfeeding	3075 (81%)	4431 (84%)	0.75 (0.37-1.51)
No pre-lacteal feeding	2939 (78%)	4223 (80%)	0.79 (0.26-2.39)
Exclusive breastfeeding	1781 (47%)	2424 (46%)	1.03 (0.70-1.52)
Continued breastfeeding	3470 (92%)	4961 (94%)	0.82 (0.33-2.04)
**Fully vaccinated children at six months**	925 (25%)	2525 (48%)	0.70 (0.30-1.65)

ANC – antenatal care, HIV – human immunodeficiency virus, PMTCT – prevention of mother to child transmissions, IPTp – intermittent preventive treatment of malaria in pregnancy, OR – odds ratio, CI – confidence interval

## DISCUSSION

We conducted a pragmatic cluster randomized trial of a globally applicable intervention package targeted at improving MNCH service use and continuum of care among mothers in Angola. We found that distribution of the MCH handbook and its supplementary interventions did not increase MNCH service utilization sufficiently enough for the attainment of the primary outcome. Our study is, to the best of our knowledge, the first to compare different types of home-based records (integrated vs non-integrated record systems) in Africa, thus adding to the limited evidence on the debate regarding the superiority of the integrated MCH handbook over stand-alone home-based records.

Our findings contradict those of a recent RCT in Bangladesh, which demonstrated significant improvement in CoC following use of the MCH handbook enhanced by mobile tools [17]. However, in their study, Gai Tobe et al. [17] leveraged the existing MCH handbook implemented in Bangladesh since 2009, and used a slightly different definition of CoC varied than we did. Regardless, we found that the MCH handbook intervention package was effective in initiating antenatal care service use, especially among women in more rural settings. Several studies have shown improvements in various elements of the continuum of maternal and child care use among mothers who reported pre- and post-natal home-based record ownership. For example, improvements in frequency of antenatal care use [18-20], delivery assisted by trained personnel, vaccination uptake for mother and child [19,20], and postnatal care guidance [21] were observed among mothers who reported ownership of MCH handbook in Indonesia [19,20], Mongolia [18], and Burundi [21]. Additionally, continuum of care was shown to increase among mothers in Ghana following the introduction of a one-page pictorial educational and record card promoting service use across the continuum of maternal and child care [22].

Preliminary findings from its pilot use prior to intervention launch [13] showed the MCH handbook was well received (unpublished results), and mothers may have actively sought to attend antenatal care to get the handbook. Consequently, the MCH handbook intervention may have worked as an incentive to modify demand-side factors in initiating antenatal care use consistent with the trial hypothesis.

The lack of significant improvement in study-defined CoC could be due to the post-hoc intra-cluster correlation being higher than expected, thus leading to our study being insufficiently powered to detect a difference. The trial sample included 10 clusters, making it sufficient for our statistical hypotheses, but relatively small, thus increasing the risk of the clusters not being similar. Simultaneously, the variation between clusters was large and the post-hoc intra-cluster correlation coefficient was estimated to be about 0.04, larger than the initially assumed value of 0.01. Under this assumption of 0.04, the power to detect the expected difference was low, at less than 50%, which is consistent with the non-significant results.

Disparities in health outcomes by urban-rural location, and inequitable access to and use of prenatal care between urban and rural areas is pervasive in Angola [9,23]. Also, the lack of human resources for health, especially in less urban areas is a major barrier to service delivery [24,25]. Although studies have shown that the use of home-based records can promote the continuum of care [4,5,17], its effectiveness is context-dependent, while availability and equitable distribution of health services are pre-requisite for effective promotion of the continuum of care [26]. In this trial’s more rural settings, travel cost and distance to facilities were frequently reported barriers for accessing MNCH services. Further, a more acute shortage of services meant that services were not always available when mothers presented at health care facilities. Thus, inadequate supply of health care services and inputs, especially in rural areas are likely to be important constraints to the attainment of the continuum of care for maternal and child health in this study. To further elucidate barriers and facilitators to the intervention delivery, we did a mixed-methods implementation study which demonstrated the need for strengthening education for health care providers [27]. Our study also highlights the importance of health care provider training. As the MCH handbook is only a tool, an important component of the MCH handbook intervention package involved health care provider training on the operations of the MCH handbook. Pre- and post-training assessments and direct observation revealed improvements in health care provider knowledge and competence in service delivery using the MCH handbook. However, urban/rural and facility level disparities in basic skills and competencies required for providing maternity services remain evident [27]. Evidence shows that health care provider training may further enhance availability and quality of maternity services when adapted to country needs [28]. Hence the need for health care providers to be sufficiently trained in the use and functions of the MCH handbook to ensure good quality care cannot be over-emphasized. Future studies should consider to include training strategies that employ evidence-based approaches such as low-dose high frequency approaches in which health care providers are trained while at work.

### Strengths and limitations

Strengths of our study include the randomized controlled trial design, comparison of the integrated MCH handbook against existing stand-alone home-based records, the rigorous trial design wherein we ensured the participant cohort had similar maternal characteristics, a low attrition rate and controlling for clustering at the municipality level in the analysis. Additionally, the MCH handbook intervention study was a population-based locally driven study done in a real-world and resource constrained setting demonstrating the practicability of the intervention.

However, it also had some limitations. First, despite the rigorous design, the study was inadequately powered. Second, as a pragmatic open-label trial, women may have actively sought maternity care in municipalities providing the MCH handbook. Similarly, we could not avoid health care provider contamination resulting from personnel postings and transfer between municipalities. The extent to which women and health care providers migrated between clusters could not be established, and if this contributed to limiting intervention impact is unclear. Incorporating some elements of the intervention package to the control group could not be fully excluded which may have led to performance bias. Other administrative barriers may have limited maternal and child health care service availability, especially in rural areas and these limitations could have affected the trial endpoint independent of the intervention package. Third, the impact of the COVID-19 pandemic on provision of MNCH services and maternal health behavior could not be established. Although the collection of follow-up data was delayed, we kept the primary outcome unchanged to account for intervention effects prior to the pandemic. Fourth, integrating the trial with routine maternity care services may have required health care providers to divert time from routine activities to complete trial related steps, which could have negatively affected implementation of the intervention package and health care service delivery. Furthermore, provision of the new service could have stretched health care provider capacity, leading to longer waiting times and hindering access for women who had to travel far to reach health facilities.

## CONCLUSIONS

Although our trial was unable to help us understand the superiority of the MCH handbook over stand-alone home-based records, we were able to show an intervention effect on initiating antenatal care use among women. This is an important finding, considering that antenatal care improves the survival and health of babies indirectly by providing an entry point for health contacts with women at a vital stage in the continuum of care. We also showed that the MCH handbook does not work in isolation and is more likely to fulfil its functional role in an improved health system. Further impact evaluation in similar settings is clearly needed to address the questions regarding superiority of the MCH handbook over stand-alone home-based records, with careful consideration for cluster selection and strategies for adequate training of health care providers.

## Additional material


Online Supplementary Document
