# Supplementary material for: Effectiveness of the maternal and child health handbook for improving continuum of care and other maternal and child health indicators: A cluster randomized controlled trial in Angola
Source: J Glob Health. 2023 Feb 3;13:04022. doi: 10.7189/jogh.13.04022 (PMC9894505; doi:10.7189/jogh.13.04022)
Supplement: Online Supplementary Document [file jogh-13-04022-s001.pdf]

ONLINE SUPPLEMENTARY DOCUMENT

Title: Effectiveness of the Maternal and Child Health Handbook for improving Continuum of Care and other Maternal and Child Health indicators: a cluster randomised controlled trial in Angola

Authors: Olukunmi Omobolanle Balogun, Ai Aoki, Caroline Kaori Tomo, Keiji Mochida, Sachi Fukushima, Masashi Mikami, Toru Sadamori, Michiru Kuramata, Freitas Reis Helga, Pedro Sapalalo, Lino Tchicondingosse, Rintaro Mori, Hirotsugu Aiga, Francisco Rubuz Ketha, Kenji Takehara

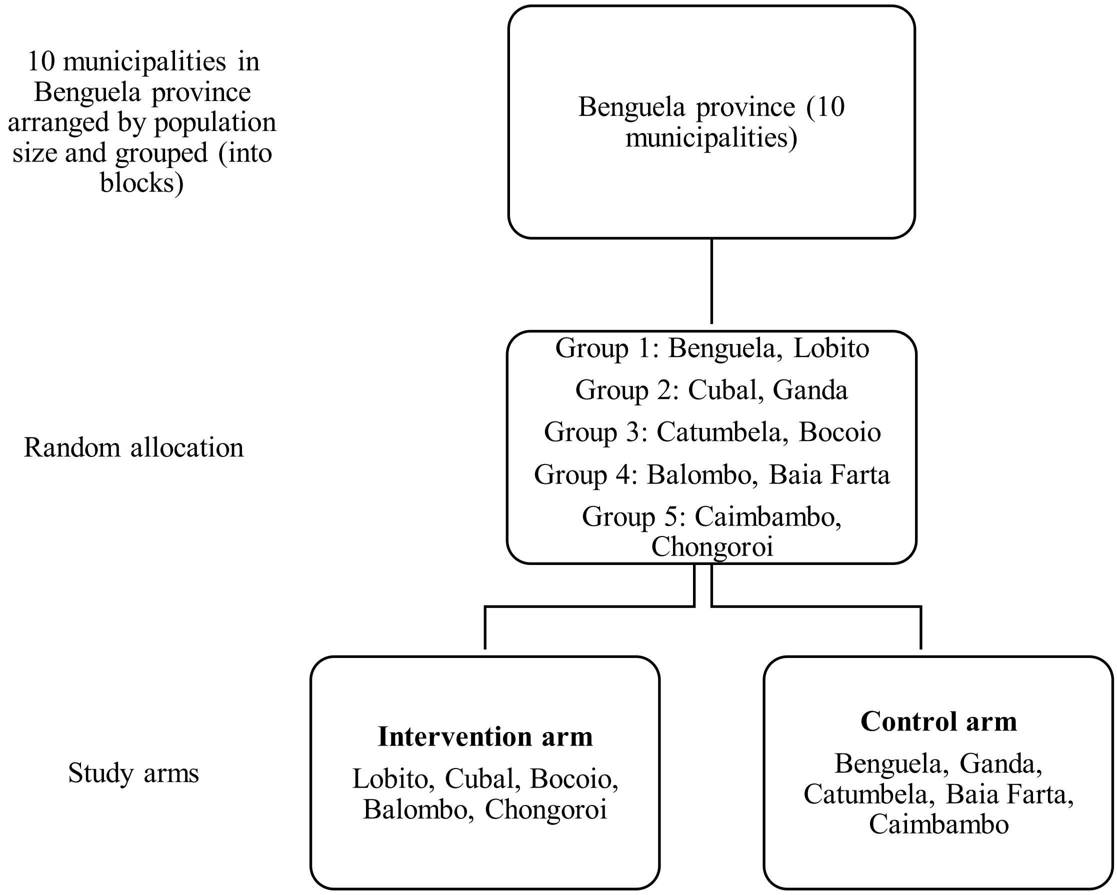

Figure S1: Cluster (municipality) allocation using block randomisation. Randomisation process was done using computer-generated results

Table S1: Power analysis for sample size estimation

Power		Intervention: 25%, Control: 15%		
Simulation: 1,000 times<br>α =0.05, Two-sided		Total N		
		5,000	7,500	10,000
ICC	0·010	0·776	0·808	0·828
	0·015	0·639	0·658	0·674
	0·020	0·560	0·570	0·605

Table S2: Analysis sets

	Study arm	Description	Distribution
ITT analysis set	Intervention	Municipality of first ANC	4,633
	Control	Municipality of first ANC	6,373
PPS analysis set	Intervention	Received MCHHb and has record	4,644
	Control	Received MC/CC and has record	6,362

ITT= intention-to-treat; PPS= per-protocol set

Table S3: Comparison of ITT analysis set and PPS analysis set

		difference		
	count	minimum	average	maximum
q124<q124pps	19	-1	-1	-1
q124=q124pps	10979			
q124>q124pps	8	1	1	1
jointly defined	11006	-1	-0·0009995	
total	11006			

q124=study arm variable (ITT and pps respectively) in analysis dataset

Table S4: Number of facilities that participated in the MCH handbook intervention trial. A comprehensive list of all registered healthcare facilities providing maternal, newborn and child health (MNCH) care services in Benguela province was obtained prior to beginning the trial. The total number of facilities listed as providing MNCH services varied slightly from number involved in the MCH handbook intervention package trial.

Municipality	No of facilities listed in Benguela province	No of facilities involved in trial	No of recruitments per municipality
Baía Farta	16	16	938
Balombo	15	14	777
Benguela	36	33	2760
Bocoio	22	17	720
Caimbambo	12	12	740
Catumbela	17	16	624
Chongoroi	16	14	632
Cubal	22	20	911
Ganda	27	24	1567
Lobito	31	24	1716
Others			14
	214	190	11399

*Others are cases where facility name was not written, or rural facilities with no name

No participants were recruited from 24 facilities either because they didn't provide MNCH services or they were not operating at the time of the study and were therefore excluded from the list of participating facilities.

Table S5: Facility type and number of recruitments from each facility type. The province's public healthcare facility network included 214 facilities across the three levels of care tertiary (provincial hospital), secondary (municipal hospitals) and primary (health center and health post). Facilities across all levels or care were recruitment points for study participants.

Municipality	Health post[a]	Health center[b]	Hospital[c]	Total
Baía Farta	300	639	0	939
Balombo	184	594	1	779
Benguela	1022	1361	378	2761
Bocoio	257	377	86	720
Caimbambo	143	344	253	740
Catumbela	193	430	6	629
Chongoroi	327	67	239	633
Cubal	579	92	240	911
Ganda	549	1012	6	1567
Lobito	561	1159	0	1720
Total	4115	6075	1209	11399

aPosto de Saúde; bCentro de Saúde Materno e Infantil/Centro de Saúde; cHospital Provincial/Municipal hospital

Table S6: Effect of MCH handbook intervention package on attainment of continuum of care by facility location. Subgroup analyses to evaluate impact of MCH Handbook for improving maternal behaviour-based CoC and service-based CoC by facility location

**Urban area**	**Intervention arm (n=1,315)**	**Control arm (n=3,299)**	**OR (95% CI)**
Maternal behaviour-based CoC	562 (43%)	1689 (51%)	1·73 (0·66-4·57)
minimum expected number of ANC	963 (73%)	2366 (72%)	1·67 (0·83-3·35)
facility based delivery	900 (68%)	2538 (77%)	1·63 (0·74-3·59)
postnatal care for mother	928 (71%)	2595 (79%)	1·60 (0·76-3·37)
postnatal care for baby	1029 (78%)	2790 (85%)	1·34 (0·63-2·87)
2 vaccination clinic visits at 3 months	872 (66%)	2619 (79%)	1·05 (0·48-2·31)
Service-based CoC	493 (37%)	1587 (48%)	1·46 (0·40-5·34)
7 vaccinations received at 3 months	710 (54%)	2344 (71%)	1·05 (0·24-4·69)
**Rural area**	**Intervention arm (n=2,451)**	**Control arm (n=1,960)**	**OR (95% CI)**
Maternal behaviour-based CoC	568 (23%)	450 (23%)	0·94 (0·29-3·03)
minimum expected number of ANC	1567 (64%)	1234 (63%)	1·23 (0·60-2·51)
facility based delivery	1043 (43%)	836 (43%)	1·03 (0·44-2·43)
postnatal care for mother	1114 (45%)	936 (48%)	0·98 (0·39-2·49)
postnatal care for baby	1413 (58%)	1353 (69%)	0·78 (0·25-2·46)
2 vaccination clinic visits at 3 months	1038 (42%)	1159 (59%)	0·56 (0·16-1·92)
Service-based CoC	495 (20%)	405 (21%)	0·80 (0·15-4·22)
7 vaccinations received at 3 months	702 (29%)	946 (48%)	0·48 (0·08-2·89)

CoC=continuum of care, ANC=antenatal care, OR=odds ratio, CI=confidence interval
